# Editorial: Hybrid or mixed myelodyplastic/myeloproliferative disorders: Current trends in diagnosis and treatment

**DOI:** 10.3389/fonc.2023.1166163

**Published:** 2023-03-08

**Authors:** Argiris Symeonidis, Ulrich Germing

**Affiliations:** ^1^ Hematology Division, Department of Internal Medicine, University of Patras, Patras, Greece; ^2^ Department of Hematology and Oncology, Heinrich Heine University of Düsseldorf, Düsseldorf, Germany

**Keywords:** mixed MDS/MPN, chronic myelomonocytic leukemia (CCML), atypical chronic myeloid leukemia (aCML), myelodyslastic/myeloproliferative neoplasms (MDS/MPN), WHO classification - myeloid neoplasms - diagnostic criteria

This Research Topic in *Frontiers in Oncology* is dedicated to the current trends in the diagnosis and treatment of hybrid or mixed myelodysplastic/myeloproliferative disorders. This group of myeloid neoplasms has not yet received enough attention in the various classifications proposed for these diseases, for several decades, been placed in the past under the umbrella of myelodysplastic syndromes (MDS) or that of myeloproliferative neoplasms (MPN).

However, it has ultimately become clear that hybrid or mixed MDS/MPN have a profile of clinical, hematologic, cytomorphologic, histomorphologic, pathogenetic, cytogenetic, and molecular characteristics, indicating that enlisting or classifying them among other disease entities, such as the typical and clearly identifiable MPN or MDS, may not be appropriate or even useful and practical. It should be emphasized that all the articles included in this Research Topic have been submitted, revised, and published before the recently published fifth edition of the World Health Organization (WHO) Classification of Myeloid Neoplasms. Thus, the authors have followed the previous fourth edition of WHO Hybrid or Mixed MDS/MPN, published in 2016, according to which five disease entities were included, namely, atypical BCR-ABL negative chronic myelogenous leukemia (aCML), chronic myelomonocytic leukemia (CMML), juvenile myelomonocytic leukemia (JMML), MDS/MPN unclassifiable (MDS/MPN-U), and MDS/MPN with ring sideroblasts and thrombocytosis (MDS/MPN-RST) as a provisional entity (1).

In the fifth edition of the WHO classification, the provisional entity MDS/MPN-RST has been confirmed as a fully, clearly, and/or molecularly identified disease, associated (although not mandatorily) with SF3B1 mutations. A new entity, namely myeloproliferative neoplasm with PCM1-JAK2 rearrangement, has been proposed, whereas JMML has been removed from this category (2). These changes have restricted the content of the so-called “Mixed MDS/MPN unclassifiable” group. Finally, another change was the recognition that a subgroup of patients with neutrophilic leukemia exhibits morphologic features of trilineage dysplasia, and, despite neutrophilia, have ineffective erythropoiesis with or without transfusion dependence. This subgroup is proposed to be classified among MDS/MPN rather than among typical MPN as it is classified as another subgroup of this condition (3). This new proposal justifies our decision to include a review article on chronic neutrophilic leukemia in the contents of this Research Topic.

In contrast to the true MPN, which can develop following the emergence of a single gene mutation, the majority of cases have a normal cytogenetic profile, are primarily characterized by an increase in peripheral blood cell counts, and are associated with thromboembolic complications and constitutional symptoms. In contrast to MDS, which usually exhibits more than one driver mutation, commonly has an abnormal cytogenetic profile, is generally associated with ineffective hematopoiesis with peripheral blood cytopenias, and shares a high risk of progression to acute leukemia, hybrid or mixed MDS/MPN may exhibit features of both hematopoietic insufficiencies with morphological aberrations and also feature the proliferation of at least one cell lineage, either of megakaryocytes or of monocytes and/or of different myeloid progenitors. In addition, these diseases can sometimes be presented with constitutional symptoms and even with thromboembolic complications. However, none of these entities included within the group of hybrid or mixed MDS/MPN is a monogenetic entity, but all of them (although to a different extent) are characterized by a variety of chromosomal and, particularly, of molecular alterations, such as somatic mutations. Moreover, none of these somatic mutations, or any combination of them, nor any chromosomal aberration is pathognomonic for the entire group of mixed MDS/MPN or even for a single member-entity of the group. An additional logistic problem is that these are very rare and inhomogeneous diseases, and therefore, large databases and biobanks, which could potentially allow us to better analyze the disease biology, are not easily available (4, 5).

This Research Topic in *Frontiers in Oncology* has therefore asked experienced hematologists, hematopathologists, and geneticists to report on the epidemiology, pathogenesis, diagnosis, classification, prognosis, and therapeutic approaches of mixed MDS/MPN. In this Research Topic, Barone et al. report that in patients with polycythemia vera (PV), the circulating megakaryocyte-derived extracellular vesicles (EV) were significantly decreased, whereas platelet-derived EVs were increased and that PV patients also had an abnormal microbial DNA signature, which can generate an abnormal inflammatory network, potentially favoring the pathogenesis of the disease. In a review, Diamantopoulos and Viniou summarize the state-of-the-art diagnostic procedures, the recently-described molecular characterization of the disease, i.e., the recurrent pathogenetic mutations, and the current treatment approaches for patients with aCML. A comprehensive review of the complex and heterogeneous molecular pathogenesis and pathophysiology of CMML, with the application of the currently available research technologies, as well as the development of potential molecular targets for the design of novel therapies is presented in an excellent article by Geissler. The rare entity of myeloproliferative neoplasm with PCM1-JAK2 rearrangement is reviewed by Sun et al., following the description of two new cases. The authors discuss the poor prognosis and the lack of treatment guidelines for this entity, as well as the unpredictable and heterogeneous response pattern of treatment with ruxolitinib. They suggest that the combination of ruxolitinib and pegylated interferon might be more effective. Fontana et al. add molecular pathogenetic considerations on BCR/ABL-negative aCML, and discuss the influence of the molecular profile of the disease on prognosis and on the potential treatment options, including the novel and emerging targeted treatments. Küendgen et al. compile the epidemiological characteristics of hybrid or mixed MDS/MPN and emphasize that with the exception of the most common disease, namely CMML, there is a lack of reliable and convincing information, mainly attributed to the rarity of these syndromes, but also to the changing classification systems and the blurry and vague limits of each of them. Liapis et al. discuss the various options for first-line treatment available to patients with advanced CMML and analyze the existing data of the two main treatment approaches, namely, cytotoxic treatment and hypomethylated agents, and how this kind of treatment can be used as a bridge for allogeneic stem cell transplantation in transplant-eligible patients. Symeonidis et al. present all the published data from a few prospective studies but mainly from retrospective studies on allogeneic stem cell transplantation for mixed MDS/MPN and analyze the prognostic factors for the various transplantation outcomes. Finally, Thomopoulos et al. complete the round of articles with an overview of the clinical characteristics, molecular genetics, and the existing and emerging therapeutic approaches for patients with chronic neutrophilic leukemia, a very rare disease with several unmet needs for appropriate diagnosis and effective treatment.

In summary, this Research Topic consists of a collection of excellent articles, aiming to help interested readers to navigate through the classifications, understand the complex pathogenesis a little bit more, and get inspiration with regard to therapeutic considerations. We believe that the readers will enjoy reading this Research Topic.

## Author contributions

AS and UG have equally contributed in the design and scientific supervision of the content of this Research Topic, and they also have both contributed to the writing of this Editorial. All authors contributed to the article and approved the submitted version.

## References

[1] Arber DA Orazi A Hasserjian R Thiele J Borowitz MJ Le Beau MM. The 2016 revision to the world health organization classification of myeloid neoplasms and acute leukemia. Blood (2016) 127(20):2391–405. doi: 10.1182/blood-2016-03-643544 27069254

[2] Khoury JD Solary E Abla O Akkari Y Allagiy R Apperly JF. The 5th edition of the world health organization classification of haematolymphoid tumours: Myeloid and Histiocytic/Dendritic neoplasms. Leukemia (2022) 36(7):1703–19. doi: 10.1038/s41375-022-01613-1 PMC9252913 35732831

[3] Arber DA Orazi A Hasserjian RP Borowitz MJ Calvo KR Kvasnicka HM. International consensus classification of myeloid neoplasms and acute leukemias: Integrating morphologic, clinical, and genomic data. Blood (2022) 140(11):1200–28. doi: 10.1182/blood.2022015850 PMC9479031 35767897

[4] Zheng G Li P Zhang X Pan Z. The fifth edition of the world health organization classification and the international consensus classification of myeloid neoplasms: Evolving guidelines in the molecular era with practical implications. Curr Opin Hematol (2023) 30(2):53–63. doi: 10.1097/MOH.0000000000000748 36728868

[5] Prakash S Arber DA Bueso-Ramos C Hasserjian RP Orazi A. Advances in myelodysplastic/ myeloproliferative neoplasms. Virchows Arch (2023) 482(1):69–83. doi: 10.1007/s00428-022-03465-7 36469102

